# Quantification of microplastics in complex environmental matrices using a tiered approach with modulated differential scanning calorimetry (MDSC)

**DOI:** 10.1007/s00216-025-06212-4

**Published:** 2025-11-24

**Authors:** Yingshu Leng, Liliana Gaburici, Xudong Cao, Shan Zou

**Affiliations:** 1 Metrology Research Centre, National Research Council Canada, 100 Sussex Drive, Ottawa, Ontario K1A 0R6 Canada; 2 Ottawa–Carleton Institute for Biomedical Engineering, University of Ottawa, 161 Louis Pasteur, Ottawa, Ontario K1N 6N5 Canada; 3 Quantum and Nanotechnologies Research Centre, National Research Council Canada, 100 Sussex Drive, Ottawa, Ontario K1A 0R6 Canada; 4 Department of Chemical and Biological Engineering, University of Ottawa, 161 Louis Pasteur, Ottawa, Ontario K1N 6N5 Canada; 5 Department of Chemistry, Carleton University, 1125 Colonel By Drive, Ottawa, ON K1S 5B6 Canada

**Keywords:** Microplastics (MPs), Modulated differential scanning calorimetry (MDSC), Thermal analysis, Quantification, Biosolid matrix, Wastewater treatment plant (WWTP)

## Abstract

The widespread presence of microplastics (MPs) in biosolids raises significant concerns, primarily because biosolids are commonly used as fertilizers in soil, where MPs can accumulate, disrupt soil health and microbial activity, and potentially enter the food chain. Accurate quantification of MPs in biosolids and soil remains challenging due to their low concentrations, aging-induced property variations, and complex biosolid matrices. To address these challenges, modulated differential scanning calorimetry (MDSC), a high-sensitivity, low-detection limit, and cost-effective thermal analysis approach, was employed to quantify MPs in complex biosolid matrices. Using micron-sized polyethylene (PE), polypropylene (PP), polyamide 6 (PA6), and polyethylene terephthalate (PET) spiked into biosolid matrices, MPs were quantified based on the enthalpies generated from the melting peaks. MDSC exhibited 1.4–2.5 times higher sensitivity than conventional DSC, with a theoretical limit of quantification (LOQ) as low as 7 μg/g. An averaged recovery of 93 ± 20% for four micron-sized plastics from three different sources using MDSC demonstrated good accuracy, confirming its reliability. To highlight its applicability to real-world samples, a tiered workflow incorporating MDSC, Raman spectroscopy, and thermogravimetric analysis (TGA) was employed to identify and quantify MPs in biosolids. These findings indicate that MDSC, especially when combined with complementary techniques, is a sensitive and accurate method for identifying and quantifying MPs in complex matrices.

**Graphical abstract:**

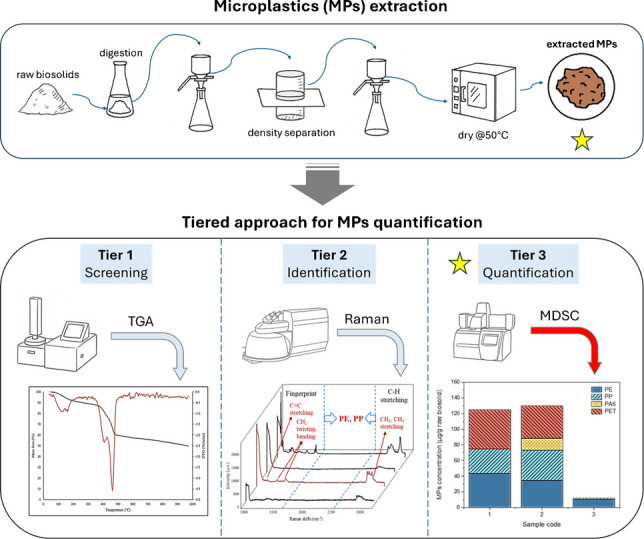

**Supplementary Information:**

The online version contains supplementary material available at 10.1007/s00216-025-06212-4.

## Introduction

Microplastics (MPs), typically defined as plastic particles smaller than 5 mm, have become a primary environmental concern due to their widespread distribution and potential to disrupt both marine and terrestrial ecosystems [1, 2]. Their presence in soil and water has raised alarms as MPs can accumulate in the food chain, potentially affecting human health and biodiversity. As MP research progresses, there is a growing focus on identifying, quantifying and understanding the environmental abundance and implications of MPs. The quantification of MPs remains particularly challenging due to their broad size distributions, diverse compositions, and complex matrices [3, 4]. Biosolids, the by-products of wastewater treatment plants (WWTPs), are major accumulators of MPs [5–7], and it has been reported that there are as many as 12,000 MP particles per kg of biosolids [8]. In North America, approximately 50% of biosolids is processed and applied as fertilizer in agriculture [9], which inadvertently makes biosolids one of the primary routes for microplastics to be released into the environment [10, 11].

As global plastic production and consumption increase, unmanaged discharges into WWTPs will likely result in more MPs entering the environment [10, 12]. Although research on microplastic pollution is advancing rapidly, identifying and quantifying MPs in biosolids remains challenging [13]. This is due to (1) difficulties in extracting MPs, (2) instrumental limitations in detecting small-sized particles, and (3) the presence of organic materials and other contaminants in matrices that complicate the quantification process [14–16]. Therefore, there is a pressing need for reliable and efficient methods to identify and quantify MPs in complex matrices, such as biosolids, to better assess their environmental risks.

Existing analytical methods, such as Fourier-transform infrared (FTIR) and Raman spectroscopies, are commonly used to identify MPs based on their chemical structures [17, 18]. While both can effectively determine the chemical characteristics of the MP samples of interest, they are often limited in quantitative measurements. While quantifications are possible through labor-intensive visual sorting or by integrating machine learning techniques, these methods frequently face challenges associated with sample heterogeneity in complex sample matrices, including biosolids [19]. Useful as they are, other techniques, such as pyrolysis gas chromatography-mass spectrometry (py-GC/MS) [20–22], are limited by high costs and interference from organic matter in the matrices, which are known to increase background noise [23]. Alternatively, thermogravimetric analysis (TGA) has also been used to quantify MPs [24, 25]. However, the TGA technique generally fails when thermal transitions overlap [26].

Differential scanning calorimetry (DSC) is a widely used technique for analyzing polymers, measuring the enthalpy changes associated with thermal transitions, primarily melting and crystallization [24, 27]. This technique allows for quantification based on the amount of heat absorbed or released [28–31]. While conventional DSC can be used to quantify polymer content in biosolids, it is limited in resolving overlapping thermal events, especially in complex samples, thus hindering its wide application in MPs analysis [28]. To address this issue, modulated differential scanning calorimetry (MDSC) has emerged as a technique that enhances detection resolution and sensitivity [32, 33]. The MDSC technique integrates conventional DSC with periodic temperature modulation, enabling simultaneous measurement responses to multiple heating rates within the same experimental setup, thereby eliminating the need for compromises between resolution and sensitivity [34]. The MDSC has been shown to enhance the analysis of MPs by separating reversible and irreversible thermal events via temperature modulation, allowing the method to distinguish overlapping thermal signals and providing a clearer understanding of complex materials [35]. This not only enhances sensitivity and resolution but also facilitates the detection of weak transitions and the separation of overlapping thermal events [33, 34, 36], making the MDSC highly effective for identifying different polymer types in complex samples and distinguishing their crystalline and amorphous phases.

In this study, we develop and validate an MDSC-based method to both identify and quantify MPs in biosolid matrices with high detection sensitivity and low LOQ. Furthermore, a tiered workflow combining MDSC, TGA, and Raman spectroscopy is proposed to enable the characterization of trace amounts of MPs, such as PE, PP, PA6, and PET, in complex matrices, including biosolids, with enhanced efficiency and sensitivity.

## Materials and methods

### Microplastics preparation and characterization

Three types of microplastics were used in this study (Table 1). Commercial microplastic powders (CMP) included PE (10 μm) and PP (42 μm) from Polysciences (Warrington, PA, USA), PET (20 μm) from Chemazone (Leduc, AB, Canada), and PA6 produced by blending Sigma PA6 pellets (Oakville, ON, Canada) and sieving through a 125 μm mesh. Product-derived microplastics (PMP) were sourced to reflect common consumer plastic waste: PE from laboratory wash bottles, PP from food containers, PET from disposable water bottles, and PA6 from umbrella straps. PE, PP, and PET were blended and sieved to < 125 μm, while PA6 straps were cut into 1–2 mm pieces and manually pulled into ~ 50 μm fibers. Aged-CMPs were prepared by suspending 50 mg of CMPs in 150 mL of 0.01% polyvinyl alcohol (PVA) solution, followed by thermal aging in an autoclave and probe sonication [37], after which the suspension was filtered through 5 μm PTFE membranes (Sartorius, Aubagne, France) and dried at 50 °C.

**Table 1:** Sources and preparation of microplastic samples for calibration and validation of the MDSC method

Source Type	Polymer	Material Description	Preparation/Size	Supplier/Origin
**Commercial Microplastic Powders** **(CMP)**	PE	Powder, ~ 10 μm	Direct use	Polysciences Inc. (Warrington, PA, USA)
	PP	Powder, ~ 42 μm	Direct use	Polysciences Inc. (Warrington, PA, USA)
	PET	Powder, ~ 20 μm	Direct use	Chemazone Inc. (Leduc, AB, Canada)
	PA6	From PA6 pellets	Blended, sieved < 125 μm	Sigma (Oakville, ON, Canada)
**Product-derived Microplastics (PMP)**	PE	Lab wash bottles	Blended, sieved < 125 μm	Consumer plastic waste
	PP	Food containers	Blended, sieved < 125 μm	Consumer plastic waste
	PET	Water bottles	Blended, sieved < 125 μm	Consumer plastic waste
	PA6	Umbrella wrist straps	Cut 1–2 mm, pulled to ~ 50 μm fibers	Consumer plastic waste
**Aged-CMP**	PE, PP, PET, PA6 (from CMPs)	Suspended in 0.01% PVA	Autoclave + sonication, filtered (5 μm PTFE), dried at 50 °C	Prepared in lab

### Microplastics extraction from biosolids

In this study, raw biosolids from a secondary-level wastewater treatment facility with anaerobic digestion were used; they were collected from various locations across Canada [5, 6]. To ensure laboratory biosafety, the collected biosolids were autoclaved before any further processing. For the pre-treatment, 5 g of raw biosolid were reacted with 150 mL of 30% H_2_O_2_ (Fisher Scientific, Ottawa, ON, Canada) at room temperature until bubbling ceased, which takes up to 7 days, indicating complete digestion. Subsequently, the mixture was filtered through a Sartorius 1.2 µm PTFE filter (Aubagne, France), and the collected solid was then transferred to a saturated CaCl_2_ solution (density of 1.42 g/cm^3^) in a custom-made glass separator [38] for density separation after the mixture was allowed to settle overnight. The CaCl_2_ solution was used because it had a higher density than most common polymers, and is considered safe and environmentally friendly. Subsequently, the supernatant– the fraction with a density < 1.42 g/cm^3^– was filtered through a 1.2 µm PTFE filter. The retained solids were collected, dried at 50 °C, and then manually ground in a ceramic mortar and pestle for 10–20 s, to de-agglomerate dried clumps prior to further analysis.

### MDSC measurement and analysis

A DSC Q2000 (TA Instruments, New Castle, DE, USA) equipped with a refrigerated cooling system (RCS90, TA Instruments, New Castle, DE, USA) was used for the MDSC measurements. Briefly, dry samples (5–15 mg) were placed into Tzero aluminum pans (TA Instruments, New Castle, DE, USA), sealed with Tzero lids (TA Instruments, New Castle, DE, USA), and then encapsulated using a Tzero DSC sample encapsulation press (TA Instruments, New Castle, DE, USA). All crucibles and sample masses were measured using a Mettler-Toledo XPE-205 analytical balance (Oakland, CA, USA) with a readability of 0.01 mg. The DSC equipment was calibrated periodically during the measurements using indium standards. Measurements were performed under MDSC mode from 50 °C to 300 °C. Specifically, during MDSC measurements, a modulated heating rate of 0.796 °C every 60 s was superimposed on a constant average heating rate, yielding total heat flow data equivalent to those obtained from conventional DSC [33]. A heat-cool-heat cycle was used to erase the thermal history and water effects and to maximize the uniformity of the crystallinity of the samples [28]. Thermograms obtained from the second heating ramp were analyzed. Samples were subjected to a heating/cooling rate of 5 °C/min to enhance the resolution of thermal transitions [39]. A nitrogen purge gas flow of 50 mL/min was maintained throughout the process.

To identify plastics in the MDSC samples, the “peak maximum” feature in the Universal Analysis 2000 (version 4.7.0.2, TA Instruments, New Castle, DE, USA) was employed to locate the peaks of melting temperatures (T_m_). This was followed by comparisons with a library of standard plastic melting peaks (Table S2) to identify potential plastic candidates.

To further quantify the amount of the identified plastics in the MDSC samples, each of the melting peak areas from the second heating cycle was integrated by applying linear peak integration at the onset and offset of the melting peaks of PE, PP, PA6, and PET at 79, 120, 168, 226, and 265 °C, respectively, all with an error of ± 1 °C. To prepare calibration curves to establish melting peak areas vs. the corresponding plastic masses, individual and varying known amounts of PE, PP, PA6, and PET CMP were pre-mixed and subsequently spiked into a digested blank biosolids (dBB) matrix, designated as CMP-dBB mixtures from hereafter. It should be noted that the "dBB matrix" refers to the biosolids obtained after digestion of the raw blank biosolid (rBB) to remove organic matter, and has been confirmed to be plastic-free via both MDSC and TGA analyses. The calibration curves were used to calculate the mass of the MP in the MDSC samples.

To evaluate potential matrix effects on polymer quantification using thermal analysis, CMP mixtures containing PE, PP, PA6, and PET were spiked into two types of environmental matrices: (1) biosolid and (2) artificial soil (AS). The samples were subjected to both conventional DSC and MDSC analysis to assess differences in thermal signal clarity. The AS matrix was a gift from Environment and Climate Change Canada, prepared in-house according to OECD guidelines, by mixing 10% sphagnum peat, 20% kaolin clay, and 70% silica sand by dry weight [40], to result in an AS with a measured organic matter (OM) content of approximately 7%.

### Determination of quantification errors in microplastics from three sources

To evaluate the accuracy and selectivity of MDSC quantification of MPs, mean absolute error (MAE) and analytical recovery were assessed using microplastic samples from three different sources –– CMP, MPP, and aged-CMP –– that were characterized using MDSC. Each sample consisted of mixtures of PE, PP, PA6, and PET in varying proportions, with each plastic ranging from 0.03 mg to 1.11 mg per sample. The resulting plastic mixtures were spiked with the dBB matrix so that the total sample mass (i.e., the plastic mix and the dBB matrix) was approximately 10 mg for the MDSC measurement. The MAE and analytical recovery were calculated using the equations below:

$$\mathrm{Mean}\;\mathrm{Absolute}\;\mathrm{Error}\;\left(\mathrm{MAE},\mathrm{mg}\right)=\frac1{\mathrm n}\sum_{\mathrm i=1}^{\mathrm n}{\vert(\mathrm m}_{\mathrm{measure},\mathrm i}{-\overline{\mathrm m}}_{\mathrm{blank}})-{\mathrm m}_{\mathrm{true},\mathrm i}\vert$$

$$\mathrm{Recovery}\;\left(\%\right)=\frac1{\mathrm n}\sum_{\mathrm i=1}^{\mathrm n}(\frac{{\mathrm m}_{\mathrm{measure},\mathrm i}-{\overline{\mathrm m}}_{\mathrm{blank}}}{{\mathrm m}_{\mathrm{true},\mathrm i}}\times100\%)$$

where ${m}_{measure}$ is the measured mass of a given species of the polymer in the spiked mixture determined by the MDSC, ${\overline{m} }_{blank}$ is the averaged peak area of the dBB matrix measured at the onset and offset of the MPs’ melting peaks (79, 120, 168, 226, and 265 °C for PE, PP, PA6, and PET, respectively); mean of six blank runs, and ${m}_{true}$ is the corresponding mass of the same polymer determined using an analytical balance (Mettler-Toledo, Oakland, CA, USA). Here, *n* is the total number of spiked samples that were measured, n = 10.

### TGA analysis

To validate the MDSC results, TGA analysis was performed using a Netzsch TG 209F1 Iris system (Selb, Germany) from 40 °C to 1000 °C at 5 °C/min under an argon atmosphere (50 mL/min). Briefly, samples (3–10 mg) were placed in an empty aluminum oxide crucible for measurement. The TGA data were processed using Proteus Analysis software (version 8.0.2, Selb, Germany), with smoothing applied to the derived thermogravimetric (DTG) curves. The decomposition temperature range of plastics (T_d_, 380–500 °C) was determined from the DTG curve, which was used to determine mass loss on the thermogravimetric (TG) curve. To assess the correlation between MDSC and TGA, different CMP mixtures containing equal amounts of PE, PP, PA6, and PET were spiked into the dBB matrix at 4%, 10% and 20% per polymer (16%, 40%, and 80% total CMP), with the remaining fraction being dBB. In each sample, TGA provided the total polymer mass, determined by the total mass loss occurring within the temperature range of 380 °C to 500 °C for the four plastics. The mass of each polymer was determined from MDSC, and the results were summed to obtain the total mass. The relative error was determined by comparing the total mass measured by the MDSC (*m*_MDSC_) with that by the TGA (*m*_TGA_), normalized to *m*_TGA_ as the reference method.

$$\mathrm{Relative}\;\mathrm{error}\left(\%\right)=\frac{{\mathrm m}_{\mathrm{TGA}}-{\mathrm m}_{\mathrm{MDSC}}}{{\mathrm m}_{\mathrm{TGA}}}\times100\%$$

### Raman spectroscopy analysis

To prepare samples for Raman analysis, 0.5 mg of the extracted materials from the tested biosolid was suspended as-is in 1 mL Milli-Q water. Then, 20 µL of the suspension was drop-cast onto a double-sided polished silicon wafer and dried in a 50 °C oven to ensure adhesion. This process was repeated three times to accumulate sufficient samples for analysis. Subsequently, Raman spectra were acquired using an inVia™ confocal Raman microscope (Renishaw plc., UK) equipped with a 633 nm excitation laser and a 1200/mm grating (50 W). Optical images were captured using a Leica 100 × objective (Concord, ON, Canada). Each Raman spectrum was recorded with a 10-s exposure time over the spectral range of 950–3200 cm⁻^1^. A total of 25 spectra were collected per sample from randomly selected different locations. The spectra were baseline corrected using a peak analyzer function in OriginPro 2021 (OriginLab, Northampton, MA, USA). For environmental samples, the obtained spectra were compared with an in-house Raman polymer spectral library for qualitative analysis.

## Results and discussion

### Microplastics characterization using MDSC and its comparison with conventional DSC

**Fig. 1:**
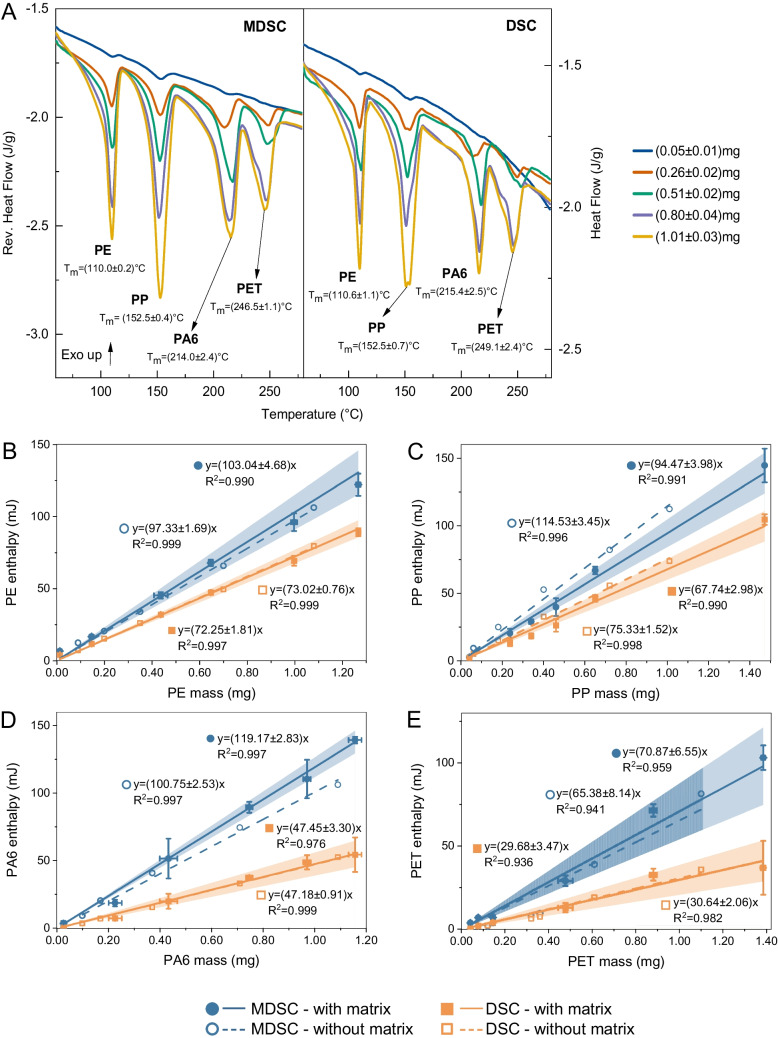
Identification and quantification of MPs by MDSC and conventional DSC. (**A)** MDSC and conventional DSC measurements of quadruple polymer mixtures spiked in biosolids. Melting peaks of polymer mixtures are shown for mixtures spiked in biosolid matrices with total masses of 0.05 ± 0.01, 0.26 ± 0.02, 0.51 ± 0.02, 0.80 ± 0.04, 1.01 ± 0.03 mg, at equal mass proportions of 25% each. Calibration curves for PE (**B**), PP (**C**), PA6 (**D**), and PET (**E**) in mixtures were obtained using MDSC and conventional DSC, with solid lines representing mixtures spiked into matrices and dashed lines representing mixtures without matrices, respectively. Calibration curves were derived from the melting peak areas (enthalpy). Fitting equations and R^2^ values are provided in each plot. Error bars indicate the standard deviation (SD) of enthalpy measurements (n = 3). Shaded confidence bands correspond to ± 2 SD, an approximate 95% confidence interval of measurement variability

It is known that MPs often exist in the environment as mixtures of various plastics and are typically embedded within complex matrices [6]. These matrices can negatively impact the accuracy of thermal analytical results, as organic and inorganic compounds within the matrices are known to lead to over- or underestimation of quantifications [41]. In the current study, we demonstrate the use of MDSC as a feasible thermal analytical method for quantifying MPs in biosolid matrices. To enhance environmental relevance and analytical accuracy, the composition of MPs in complex matrices was mimicked by spiking the dBB matrices with mixtures of CMP-PE, CMP-PP, CMP-PA6, and CMP-PET. As shown in Fig. 1A, distinct melting peaks corresponding to the semicrystalline phases of PE, PP, PA6, and PET were identified, allowing clear differentiation in both MDSC and conventional DSC.

Closer analysis of the thermograms revealed that at low plastic concentrations (i.e., 0.05 mg), MDSC exhibited a flatter baseline and more distinct peak shapes than conventional DSC, particularly for PA6 and PET. This observation is likely due to MDSC’s enhanced ability to detect weak thermal transitions by separating overlapping transitions from matrix decomposition and plastic melting [33, 42, 43], which provides a basis for quantifying MPs in complex biosolid matrices.

The thermal behaviors of individual CMPs (i.e., CMP-PE, CMP-PP, CMP-PA6, and CMP-PET) were separately characterized using the MDSC. Distinct melting points were observed at approximately 110.5 ± 0.7 °C, 155.1 ± 0.6 °C, 220.2 ± 0.4 °C, and 239.5 ± 0.3 °C for CMP-PE, -PP, -PA6, and -PET, respectively (Figure S1). It is interesting to note that for a given polymer, differences in melting point were observed when the polymer was characterized as a polymer mixture vs. individually by MDSC. This was particularly pronounced for PA6 and PET, with melting points shifting up to 7 °C and 8 °C, respectively (Figures S1C–D). A similar T_m_ shift in PA was also observed in a previous study [29]. Additionally, it is consistent with a recent TG study on mixed thermoplastics; PA6 and PET pairs exhibit interfacial interactions that accelerate degradation and deviate from linear superposition, particularly when contact is high, indicating that phase interactions can alter thermal behavior in mixtures [44]. The shift in melting peaks, therefore, justifies the importance of using polymer mixtures — as opposed to individual polymers — in MPs studies to better understand polymer behavior and achieve more accurate analytical results.

It is noticeable that as the mass of polymers increased, the peak area observed in the MDSC thermograms increased proportionally. To achieve quantification, the reversed heat flow resulting from the plastics' melting behavior during the second heating ramp was utilized, as the melting of plastics is a reversible thermal behavior, to integrate the area under each peak. Additionally, the total heat flow from the second heating ramp was generated and used as a comparative measure, equivalent to conventional DSC.

Building on this proportional relationship, calibration curves were generated by integrating peak areas under the melting peaks and plotting them against the corresponding masses of each plastic, using CMP mixtures prepared with and without dBB matrices. Figure 1B–E present the calibration curves for PE, PP, PA6, and PET individually, obtained during the second heating ramp by both MDSC and conventional DSC. All curves showed coefficients of determination (R^2^) greater than 0.99, except for PA6 in matrices and for PET (with or without matrices), analyzed by conventional DSC, which exhibited lower R^2^ values in both MDSC and DSC analyses. This is most likely attributed to the secondary reaction involving PA6 in the presence of PET during co-heating, an observation well documented in previous studies [45]. Consistently, it has been reported that PET and PA6 show the highest deviations in recovery and the most significant relative standard deviations. Moreover, the calibration curve using PET alone spiked into dBB matrices yielded a good linear fit (R^2^ > 0.99; Figure S2) in the absence of PA6, further supporting the notion that such secondary reactions affect quantitative reliability when analyzing mixtures containing these polymers.

A closer analysis of the calibration curves reveals that the dBB matrices did not affect the results from either MDSC or conventional DSC when the second heating ramp of the heat–cool–heat cycle was used, as evidenced by the similar slopes of curves with and without dBB matrices. However, as the compositions of matrices from different sources vary, potential matrix effects must still be carefully considered. Thus, calibration curves generated with biosolid matrices were used for all subsequent quantification.

### Higher sensitivity and lower LOQs of MDSC

The sensitivity of MDSC and conventional DSC for plastic quantification was compared using the slopes of calibration curves [46]. MDSC exhibited consistently steeper calibration slopes across all polymers (Fig. 1B–E), suggesting a sensitivity 1.4–2.5 times higher than that of the conventional DSC method for all polymer types investigated. This increased sensitivity arises primarily from MDSC’s ability to detect subtle enthalpy changes associated with metastable crystallites that can form due to previous thermal or mechanical processing or other pre-treatments of the plastics [47]. Such enhanced detection is particularly beneficial for low-mass samples or those with weak thermal responses that might otherwise go undetected by conventional DSC.

The limit of quantification (LOQ) for each polymer was determined from the calibration curves using the standard approach: $LOQ=\frac{10 \times SD}{S}$, where *SD* is the standard deviation of the peak area of the blank (dBB matrix) measured at the onset and offset of the MPs’ melting peaks (79, 120, 168, 226, and 265 °C for PE, PP, PA6, and PET), and *S* is the slope of the calibration curve [48]. Using this approach, MDSC achieved lower theoretical LOQs than conventional DSC for all polymers, reaching as low as 0.0001 mg per measurement, corresponding to ~ 7 μg/g in concentration for PA6 (Table S1), confirming its higher sensitivity.

**Fig. 2:**
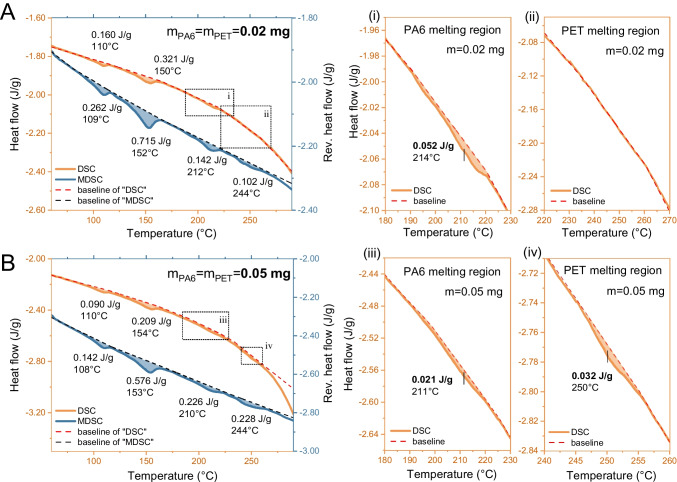
MDSC and conventional DSC thermograms of polymer mixtures spiked in digested blank biosolid (dBB). PA6 and PET were added in equal amounts at two total masses: (**A)** 0.02 mg and (**B)** 0.05 mg. Insets (i–iv) show enlarged views of the PA6 and PET melting regions from the conventional DSC thermograms

For example, at a PET sample mass of 0.02 mg (Fig. 2A), the PET melting peak at 244 °C was clearly detectable in the MDSC thermogram. In contrast, no corresponding peak was observed in conventional DSC (Fig. 2A, ii) until the sample mass was increased to ≥ 0.05 mg (Fig. 2B, iv). It is noted that PA6 shows a higher melting enthalpy at the lower mass (Fig. 2A, i and B, i). This is because, with a fixed PA6:PET ratio (1:1), increased PET content enhances PET’s cold crystallization and the leading tail of its melt, which affects the baseline near the PA6 melting point. The lower detection threshold of MDSC is likely due to its slower underlying heating rate, which keeps the system closer to equilibrium and provides a more stable baseline for detecting weak thermal transitions [49]. In addition, MDSC can separate reversible polymer melting from non-reversible signals arising from matrix decomposition [33, 43], allowing more sensitive detection of small amounts of plastics in complex matrices such as biosolids, where incomplete decomposition of inorganic matter may otherwise obscure weak melting peaks in conventional DSC.

It should be mentioned that although the LOQs of MDSC are higher than those achieved by mass spectrometry-based methods, the lowest theoretical LOQ for PA6 at 7 μg/g is comparable to that of the Py-GC/MS [50]. While MDSC measurements require larger sample sizes (generally > 0.1 mg of dried solids per measurement), the increased sample size reduces heterogeneity in sampling, thus lowering variability in results from heterogeneous samples such as MPs in biosolids.

By isolating plastic melting signals and performing matrix decomposition, MDSC improves sensitivity for plastic quantification. In the environmental-mimicked scenario with CMP mixtures in biosolid matrices, these low quantification limits enable the detection of MPs at trace levels. Compared to conventional DSC, MDSC provides more reliable measurements with smaller sample sizes, offering advantages for detecting increasingly pervasive MPs at minute concentrations.

### Factors affecting sensitivity and accuracy in MDSC quantification

#### Crystallinity and aging history

Quantifying low-crystallinity or amorphous plastics by conventional DSC is challenging because their thermal responses are weak or diffuse, lacking the sharp melting transitions and large enthalpy changes typical of highly crystalline polymers [51]. For instance, polystyrene (PS) undertakes a glass transition rather than melting. As illustrated in Figure S3, MDSC can detect the presence of PS. Still, the signal-to-noise ratio is substantially reduced, particularly in multi-component mixtures with PE, where the melting of low-density PE (LDPE) overlaps with the PS glass transition [30]. Among the four micron-sized plastics analyzed, PE and PP displayed the highest thermal response (Fig. 1A), owing to their defined crystallinity [28, 52]. In contrast, PET exhibited the weakest signal, consistent with its lower crystallinity fraction [28, 53].

Natural aging processes, such as UV radiation, mechanical abrasion, and chemical oxidation, often result in reduced crystallinity [54, 55], thereby diminishing thermal signals [56]. Additionally, manufacturing conditions and the presence of additives can also modify polymer crystallinity structures and affect enthalpy, resulting in reduced accuracy and sensitivity [57].

To explore how different manufacturing and aging histories affect quantification sensitivity, calibration curves were generated using micron-sized mixtures of PE, PP, PA6, and PET from the two sources of microplastics introduced earlier, PMP and aged-CMP, each spiked separately into the dBB matrix. As shown in Figure S4, the PMP set yielded the lowest calibration slopes, with PMP-PP being 59% less sensitive than CMP-PP. This reduction likely originated from broader, weaker melting peaks arising from lower crystallinity after manufacturing [58, 59]. Although the heat-cool-heat cycle (in non-isothermal crystallization) can partially restore crystallinity through recrystallization during cooling, this effect depends on polymer-specific kinetics that are difficult to control uniformly in mixed-plastic samples [28, 60]. Despite these challenges, MDSC retained good sensitivity in quantifying trace micron-sized plastics with varying aging and manufacturing history, by capturing weak transitions more effectively.

**Fig. 3:**
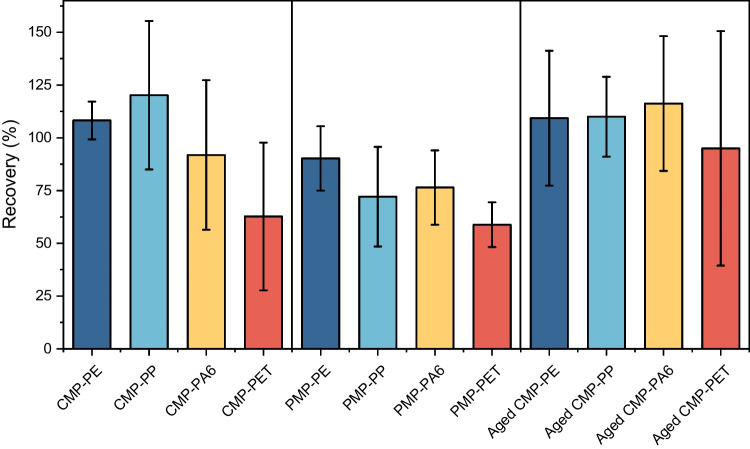
Validation of quantification accuracy using CMP, PMP, and aged-CMP. Spike recoveries (%) of PE, PP, PA6, and PET measured by MDSC. Bars show mean recovery and error bars denote standard deviation (SD) of 10 independent spiked samples (n = 10).

To evaluate the accuracy of MDSC for microplastics quantification, the measured mass from MDSC (m_*measure*_) was compared with the actual weighed mass (m_*true*_). Three mixtures—CMP, PMP, and aged-CMP (Table 1)—are each spiked into the dBB matrix at concentrations ranging from 0.03 to 1.11 mg per plastic, with 10 replicates per mixture (n = 10). Specifically, the m_*true*_ values are obtained gravimetrically, while m_*measure*_ values are determined by MDSC from melting peak areas and converted to mass using calibration curves. Comparisons between MDSC and gravimetric values are shown in Figure S5, and the mean absolute errors (MAE) are summarized in Table 2. The analytical recovery of MDSC quantification for PE, PP, PA6, and PET is presented in Fig. 3.

**Table 2:** Deviation of MDSC-measured microplastic mass from theoretical values for CMP, PMP, and aged-CMP. Values are mean ± standard deviation over n = 10 independent spiked samples per mixture

Plastic types	Mean absolute errors (mg)		
	CMP	PMP	aged-CMP
PE	0.02 ± 0.01	0.03 ± 0.02	0.02 ± 0.01
PP	0.04 ± 0.02	0.18 ± 0.13	0.05 ± 0.03
PA6	0.02 ± 0.02	0.06 ± 0.04	0.03 ± 0.02
PET	0.04 ± 0.03	0.13 ± 0.08	0.06 ± 0.05

Among the four plastics, PE exhibited the closest agreement across all sources (Figure S5A), with MAE consistently below 0.03 mg, and recovery averaged at 102% (Table 2, Fig. 3). This likely reflects its favorable crystallization kinetics, resulting from a higher molecular weight and fewer branching points, which support the formation of a stable lattice structure and a better-defined thermal transition [61, 62]. Conversely, PP showed underestimations at higher masses (Figure S5B)––when PP was spiked more than 0.4 mg in the matrices––particularly notable in PMP-PP, with an MAE of 0.18 mg, and recovery of 72%. A similar underestimation trend was observed in PA6 and PET (Fig. 3 and Figure S5 C-D), which consistently exhibited underestimations across the entire mass range in PMP, with recovery of 76% and 59%, respectively.

Regarding the aging effect, oxidative aging treatment induced chemical changes in the aged-CMP-PE, PP, and PA6 compared to CMP, as presented in the Fourier-transform infrared spectroscopy with attenuated total reflectance (FTIR-ATR) spectra in Figure S6. In aged CMP-PP and -PA6, a carbonyl peak around 1720 cm^−1^ emerged, corresponding to C = O stretching and serving as a typical indicator of MPs oxidative aging [63, 64]. The spectral changes in aged-CMP-PE were more pronounced, with a new peak around 1200 cm^−1^ suggesting the formation of new interchain interactions among PE chains due to thermal aging [65]. Although the chemical characteristics changes have been observed in PE, PP, and PA6 in aged-CMP, the measurement errors of aged-CMP did not parallel the error increment seen in PMP, especially for PP and PET. Slight overestimations were noted for PP (MAE of 0.05 mg, recovery of 110%) and PA6 (MAE of 0.03 mg, recovery of 116%), which may be due to increased crystallinity after oxidation. Since the calibration is based on the reversing melting enthalpy, which is proportional to crystallinity, the higher crystallinity of aged plastics could explain the overestimation of quantification. This observation also aligns with previous reports that oxidized PP exhibits higher crystallinity than the unoxidized ones because chemical crystallization occurs during oxidation [66, 67]. Similarly, for PA6, the oxidation enhances crystallinity, as low-molecular-weight oxidation products promote crystallization at elevated temperatures [68].

Overall, CMP mixtures consistently achieved the highest accuracy, as determined by both MAE and recovery, followed by aged-CMP, with PMP showing the largest deviations. These discrepancies may arise from additives or contaminants introduced during manufacturing, which interfere with chain mobility and disrupt crystallization [69–72]. This trend suggests that both manufacturing and post-consumer modifications can complicate crystallinity and, in turn, the precision of MDSC quantification. While PMP was consistently underestimated, variations introduced by additives and contaminants are inevitable factors that could be present during the manufacturing process. Despite these challenges, this study establishes a foundation for applying MDSC in quantifying MPs and successfully demonstrates its efficacy and potential for more precise quantification across diverse types of plastics and sources.

#### Matrix interference in environmental samples

Although the second heating ramp is commonly used to minimize matrix interference [28, 29], this approach is not always suitable for measuring plastic mixtures within complex matrices. In some cases, matrix–polymer interactions prevent recrystallization, leading to inaccurate quantification or missing signals of plastics during the second heating. For example, PA6 in a CMP mixture spiked into artificial soil (AS) failed to show a melting peak during the second heating. In contrast, the same polymer in digested biosolid did (Figure S7), suggesting that the AS matrix suppresses recrystallization. A possible cause is interfacial interactions between kaolinite and PA6 (e.g., hydrogen bonding between kaolinite surface -OH groups and PA6 amide groups), which restrict chain mobility and thereby inhibit second-heat recrystallization of PA6 [73]. Such cases highlight the importance of evaluating the first heating profile. Considering matrix variability, understanding the matrix and incorporating it as a background when establishing calibration curves is essential.

**Fig. 4:**
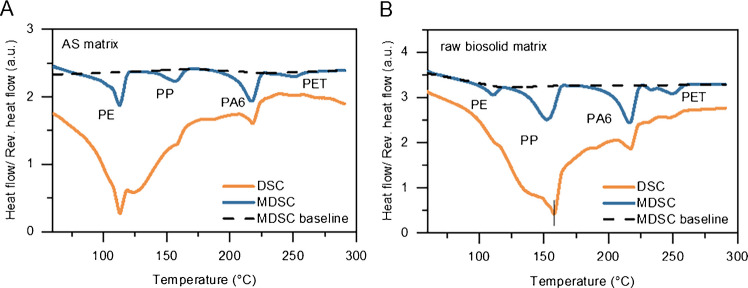
Comparison of DSC and MDSC thermograms for microplastic mixtures in matrices. Thermograms from the first heating ramp are shown for mixtures spiked into (**A) **artificial soil (AS) and (**B)** raw biosolids. Dashed lines indicate the baseline levels in MDSC, highlighting improved resolution and separation of polymer melting transitions compared with conventional DSC

Because matrix effects can influence thermal behavior during the second heating, ensuring a flat baseline during the first heating ramp is critical. To assess the impact of the matrix on MDSC quantification, CMP mixtures of PE, PP, PA6, and PET were spiked into raw biosolid and AS, and the samples were analyzed using both MDSC and conventional DSC. As shown in Fig. 4, the MDSC thermograms of the first heating ramp displayed smooth baselines and clearly identifiable peaks, compared with those of conventional DSC. This improvement stems from MDSC’s ability to distinguish between reversible polymer melting and irreversible matrix degradation, thereby minimizing matrix effects. With this capability, extensive sample pre-treatment can be reduced, streamlining processing while maintaining analytical accuracy.

Taken together, these findings underscore MDSC's capacity to address two persistent obstacles in the thermal analysis of MPs: weak transitions in low-crystallinity plastics and matrix interference in environmental samples. The technique provides a streamlined, robust approach for quantifying microplastics with diverse thermal properties across diverse matrices.

### Practical strategy for MP identification and quantification: a tiered approach

When analyzing environmental samples, the diverse and complex nature of MPs necessitates combining multiple analytical techniques for accurate characterization. Due to their varying sizes, shapes, and chemical compositions, relying on a single method often falls short of providing a comprehensive assessment [4, 74]. Recognizing these challenges, we propose a tiered approach to analyze MPs in environmental samples, integrating Raman and TGA as complementary techniques for MDSC identification and quantification.

**Fig. 5:**
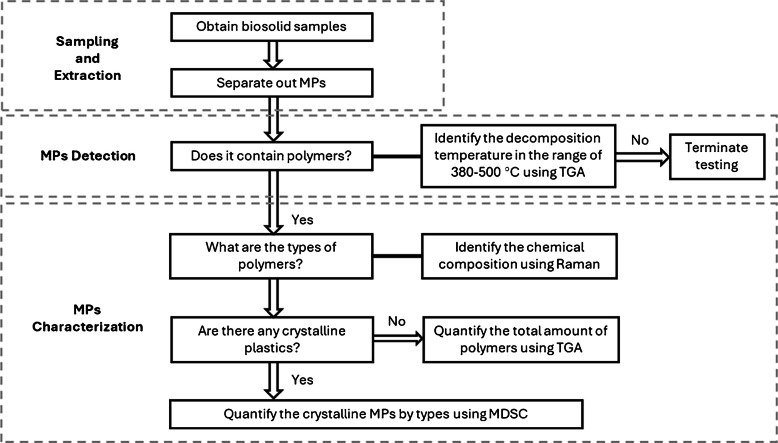
Tiered analytical workflow for microplastic (MP) analysis in biosolids. Samples are screened for polymer presence using TGA, followed by polymer type identification with Raman spectroscopy. Crystalline MPs are quantified by MDSC, while TGA provides total polymer content, enabling complementary and comprehensive characterization

Figure 5 illustrates the proposed workflow for MPs analysis in biosolids or soils. This approach begins with sample collection and separation of MPs. Initially, the presence of plastics in the samples is determined by TGA, which analyzes the degradation behavior within the typical polymer degradation temperature range (380–500 °C). Once the presence of polymers is confirmed, Raman spectroscopy is used to identify polymer types via characteristic vibrational modes that thermal methods alone cannot provide.

To determine which polymers are suitable for MDSC quantification, crystalline identity is assessed either through crystallinity-sensitive peaks in Raman spectra [75, 76], where applicable, or inferred from established material properties, since many common MPs such as PE, PP, and PET are semicrystalline under ambient conditions. Crystalline plastics can then be quantified individually using MDSC, which provides polymer-specific measurements even in the presence of complex matrix residues.

It is important to note that MDSC cannot reliably quantify amorphous plastics, such as polystyrene (PS), due to the overlap of glass transition signals with other thermal events and the lack of a sharp melting peak. In such cases, TGA and Raman are particularly valuable: TGA provides total polymer mass, including amorphous MPs [24, 30, 77], while Raman confirms their identity. This combination of MDSC and TGA could enhance understanding of MP analysis and address challenges in using MDSC to quantify non-crystalline plastics, particularly in multi-component MPs embedded in complex environmental matrices. Thus, the tiered approach leverages the strengths of each technique: MDSC resolves trace amounts of crystalline MPs with high sensitivity, Raman provides molecular specificity, and TGA complements the workflow by detecting amorphous or otherwise indistinguishable plastics.

Correlation experiments were conducted to assess the consistency and complementarity of TGA and MDSC in quantifying crystalline plastics. The CMP-dBB mixtures with PE, PP, PA6, and PET, in equal proportions of 4%, 10%, and 20% per polymer (16%, 40%, and 80% total CMP), demonstrated the complementarity of MDSC and TGA across the low–high concentration range. A 1:1 reference line was used as a benchmark to evaluate the correlation between the methods (Figure S8). At a higher mass ratio, MDSC and TGA agreed within 7% relative error. In contrast, at lower masses, the relative error increased to − 31% to − 55%, due to a broader thermal degradation range captured by TGA, which accounts for all types of polymer decompositions within 380–500 °C. At the same time, MDSC primarily resolves well-defined thermal transitions. Additionally, matrix interference could contribute to the overestimation of TGA, potentially amplifying the discrepancy between MDSC and TGA results. Overall, this validates the use of TGA as a complementary technique to MDSC for comprehensive analysis of MPs.

By explicitly combining MDSC, Raman, and TGA, this tiered approach accounts for both crystalline and amorphous plastics, compensates for matrix and aging effects, and provides a more robust and reliable workflow for quantifying MPs in complex environmental matrices [78, 79].

### Application of MDSC quantifying microplastics in biosolids

To advance the quantification of MPs in real-world biosolids, a tiered approach incorporated with MDSC was applied to analyze MPs extracted from three raw biosolids collected from different WWTPs. While MDSC is inherently less sensitive to matrix interferences than spectroscopic methods, extraction by digestion (to remove large amounts of cellulose that can hinder polymer identification) and density separation (to eliminate higher-density matter) further simplify the sample and provide a more consistent basis for accurate quantification.

Thermal decomposition profiles of the extracted biosolid, as determined by TGA measurements (Figure S9), revealed contributions from organic matter (200–400 °C), cellulose (300–400 °C), polymer decomposition (380–500 °C), and inorganics (> 500 °C). In Sample 1, a broad degradation peak spanning 250 °C to 550 °C, including a strong feature at 455 °C, suggested plastic degradation but overlapped with other components (Table S2), making it difficult to distinguish individual plastic types. Additionally, the shoulder peak around 355 °C was likely due to residual cellulose from H_2_O_2_ digestion or plastics that have undergone processing or environmental weathering [80, 81], which reduced their thermal stability, leading to lower decomposition temperatures. However, confirming the precise origin of this peak by TGA is challenging. Similar degradation patterns were noted in Samples 2 and 3, which displayed similar behaviors, with a cellulose-related peak at ~ 345 °C and broad polymer signals between 380 and 500 °C. Overall, the TGA confirmed the presence of polymer(s) but lacked the resolution to distinguish specific plastic types.

**Fig. 6:**
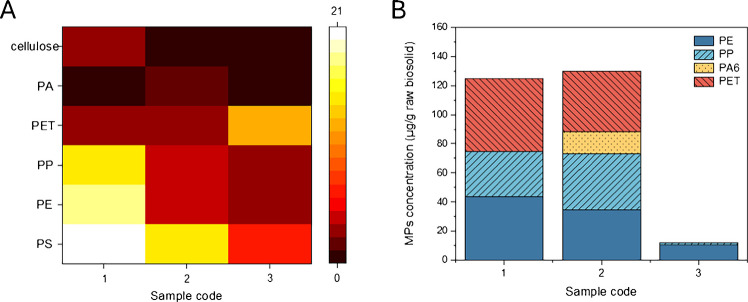
Integrated spectroscopic and calorimetric analysis of microplastics in biosolids. (**A)** Heat map showing the frequency of occurrence of polymers (or cellulose) across three biosolid samples. Occurrence frequency is defined as the number of Raman spectra in which characteristic peaks of polymers were identified. The color scale (0–21) represents the Raman spectral counts assigned to each polymer type. (**B)** Concentration of MPs extracted from real-world biosolids quantified by MDSC

Following the tiered approach workflow (Fig. 5), Raman spectroscopy was used to determine the chemical structures and identify specific polymer types. By comparison with a reference library of Raman spectra previously established from standard plastics, the presence of PS, PE, PP, and PET was confirmed in all three samples. Additionally, cellulose was observed in Sample 1, and PA was observed in Sample 2. A heatmap of the spectral counts (Fig. 6A) provided a qualitative and semi-quantitative overview of the polymer distributions across samples.

For crystalline plastics—PE, PP, PA6, and PET—MDSC provided quantitative results (Fig. 6B). As shown in Fig. 6B, Samples 1 and 2 showed comparable total concentrations of MPs, at 125 ± 48 μg/g and 130 ± 72 μg/g, respectively, suggesting a similar overall level of MPs contamination in these biosolid sources. Their compositions, however, differed. In Sample 1, PE, PP, and PET were present at concentrations of 44 ± 30 μg/g, 31 ± 11 μg/g, and 50 ± 41 μg/g, with PET contributing the largest share. Sample 2 contained a broader mix, with PA6 detected exclusively at 15 ± 5 μg/g, followed by PET (42 ± 18 μg/g), PE (35 ± 15 μg/g), and PP (39 ± 55 μg/g) together accounted for most of the MPs. By contrast, Sample 3 exhibited markedly lower levels of MP presence, with only PE and PP quantified at 4 ± 2 μg/g and 2 ± 2 μg/g, giving a total of 12 ± 4 μg/g. The relatively larger standard deviations (15–55 μg/g) observed in Samples 1 and 2 likely stem from the intrinsic heterogeneity of biosolids, which results in uneven MP distribution. Despite this variability, the MDSC results followed the same trends as the Raman identifications, as shown in Fig. 6A, reinforcing the reliability of MDSC quantification in complex matrices. As for PS, although it is consistently detected by Raman, it cannot be quantified by MDSC due to its amorphous nature and the overlap with PE melting, which prevents clear resolution under current conditions.

TGA-based estimates, obtained from mass loss within the 380–500 °C temperature range (Figure S9), were consistently higher than those from MDSC (Table S3). The discrepancy was most pronounced in Sample 3, where TGA values were nearly 75-fold higher, likely indicating substantial amounts of amorphous plastics not captured by MDSC, as well as possible effects of uneven polymer distribution in the heterogeneous biosolids.

Overall, the combined application of TGA, Raman spectroscopy, and MDSC provided complementary insights into microplastic characterization: TGA confirmed the presence of polymers and overall polymer content, Raman spectroscopy identified polymer types, and MDSC quantified the crystalline fractions. The consistency between Raman and MDSC enhanced confidence in the quantification, while comparison with TGA underscored the additional contribution of amorphous plastics. Together, this integrated approach establishes a comprehensive framework for the analysis of microplastics in complex biosolid matrices.

## Conclusions

This study established a method for quantifying MPs using MDSC in complex biosolids. By incorporating periodic temperature modulation, MDSC enhances conventional DSC by increasing sensitivity, reducing detection limits, and facilitating the separation of weak plastic melting transitions from matrix degradation. The capability provides a smooth baseline during the first heating cycle, enabling accurate differentiation of polymer signals from complex biosolid matrices. A key observation was that PA6 recrystallization could be suppressed by the matrix, potentially leading to no detectable signal during the second heating, thereby necessitating characterization during the first heating via MDSC.

Calibration curves for PE, PP, PA6, and PET were established by integrating melting peak areas relative to mass, each showing strong linearity. Potential sources of quantification error were investigated by using three different types of MPs—CMP, MPP, and aged-CMP—and all confirmed the accuracy of MDSC measurements. The method was then employed to analyze MP concentrations in biosolids, where, within the proposed tiered workflow, Raman spectroscopy identified polymer types and TGA verified polymer signals in the thermal range. These complementary techniques supported the MDSC quantification, and the results were consistent across all three biosolid samples, successfully confirming their MP concentrations.

This study demonstrated that MDSC provides high sensitivity, low detection limits, and reliable quantification of trace and low-crystallinity MPs in biosolids. Its application is currently limited to crystalline and semicrystalline plastics, and further research is required to reduce signal-to-noise ratios and resolve glass transition and peak overlaps to allow quantification of amorphous polymers.

## Supplementary Information

Below is the link to the electronic supplementary material.

Supplementary Material 1 (DOCX 606 KB)

## Data Availability

All data generated or analyzed during this study is included in the paper and will be available upon request.
