# Supplementary material for: Quantification of microplastics in complex environmental matrices using a tiered approach with modulated differential scanning calorimetry (MDSC)
Source: Anal Bioanal Chem. 2025 Nov 24;418(1):203–17. doi: 10.1007/s00216-025-06212-4 (PMC12775130; doi:10.1007/s00216-025-06212-4)
Supplement: Supplementary file 1 — Supplementary Material 1 (DOCX 606 KB) [file 216_2025_6212_MOESM1_ESM.docx]

**Quantification of Microplastics in Complex Environmental Matrices Using a Tiered Approach with Modulated Differential Scanning Calorimetry (MDSC)**

Yingshu Leng ^1,2^, Liliana Gaburici ^3^, Xudong Cao ^2,4^*, Shan Zou ^1,5^*

^1^ Metrology Research Centre, National Research Council Canada, 100 Sussex Drive, Ottawa, Ontario K1A 0R6, Canada

^2^ Ottawa–Carleton Institute for Biomedical Engineering, University of Ottawa, 161 Louis Pasteur, Ottawa, Ontario K1N 6N5, Canada

^3^ Security and Disruptive Technologies Research Centre, National Research Council Canada, Ottawa, Ontario K1A 0R6, Canada

^4^ Department of Chemical and Biological Engineering, University of Ottawa, 161 Louis Pasteur, Ottawa, Ontario K1N 6N5, Canada

^5^ Department of Chemistry, Carleton University, 1125 Colonel By Drive, Ottawa, ON K1S 5B6, Canada

*Corresponding Author: Prof. Xudong Cao, Phone: 1-613-562-5800 ext. 2097, Email: [xcao@eng.uottawa.ca](mailto:xcao@eng.uottawa.ca)

*Corresponding Author: Dr. Shan Zou, Phone: 1-613-949-9675, Email: [Shan.Zou@nrc-cnrc.gc.ca](mailto:Shan.Zou@nrc-cnrc.gc.ca) (ORCID: 0000-0002-2480-6821)

**Table S1.** The theoretically calculated limit of quantification (LOQ) for polyethylene (PE), polypropylene (PP), polyamide 6 (PA6), and polyethylene terephthalate (PET) was determined by MDSC and conventional DSC.

|  | MDSC | | Conventional DSC | |
| --- | --- | --- | --- | --- |
|  | per measurement (mg) | (μg/g) | per measurement (mg) | (μg/g) |
| PE | 0.0003 | 22 | 0.0004 | 30 |
| PP | 0.0003 | 26 | 0.0005 | 44 |
| PA6 | 0.0001 | 7 | 0.0038 | 310 |
| PET | 0.0004 | 37 | 0.0171 | 1410 |

**Table S2.** Melting point (T_m_) and decomposition temperature (T_d_) of plastics measured by DSC and thermogravimetric analysis (TGA).

| Plastic types | T_m_/°C | T_d_/°C | Reference |
| --- | --- | --- | --- |
| Polystyrene (PS) | - | 420 | - |
| Low-density polyethylene (LDPE) | 111 | 470 | - |
| High-density polyethylene (HDPE) | 139 | 478 | [1] |
| Polypropylene (PP) | 155 | 460 | - |
| Polytetrafluoroethylene (PTFE/Teflon) | 318 | 565 | - |
| Polyethylene Terephthalate (PET) | 239 | 435 | - |
| Polyamide 6 (PA6) | 220 | 420 | - |
| Polyvinyl chloride (PVC) | - | 300, 467 | - |

**Table S3.** Concentration of polymers extracted from real-world biosolids quantified by TGA.

| Sample code | Sample 1 | Sample 2 | Sample 3 |
| --- | --- | --- | --- |
| Concentration of polymers (mg/g) | 5.53 | 1.47 | 1.40 |

**Figure S1.** MDSC thermograms of individual commercial microplastic powder (CMP). (A) PE, (B) PP, (C) PA6, (D) PET.


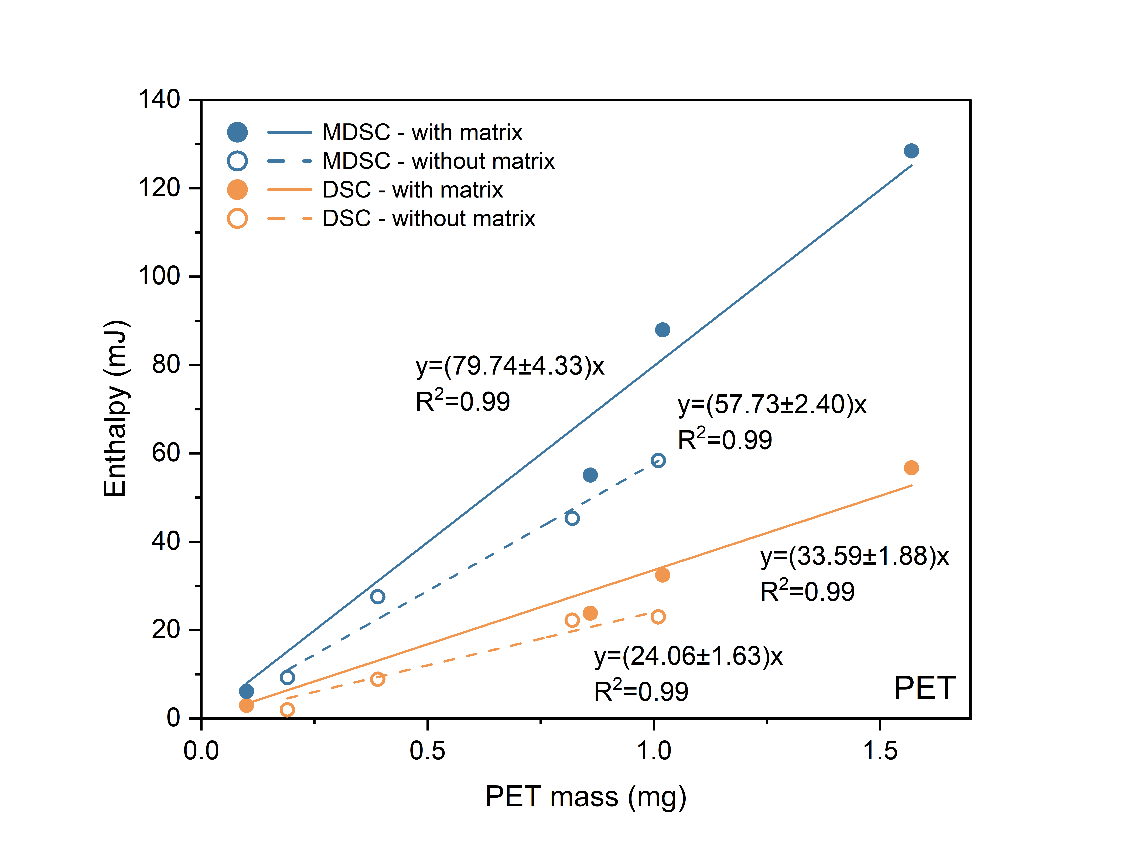


**Figure S2.** Calibration curves for PET CMP.

**Figure S3.** Total heat flow curves (black, bottom) and 1^st^ derivative of the total heat flow (red, top) of the PS and PE mixture (1:1 mass ratio) spiked in digested blank biosolid (dBB) obtained using DSC.

**Figure S4.** Calibration curves for (A) PE, (B) PP, (C) PA6, (D) PET product-derived microplastics (PMP) and Aged-CMP in the mixtures with dBB matrices. Error bars indicate the SD of the enthalpy measurements (n=3).

**Figure S5.** Validation of the accuracy of the quantification method by using (A) PE, (B) PP, (C) PA6, (D) PET CMP, PMP, and Aged-CMP. The dashed line serves as a 1:1 reference. (E) Mean absolute error of PE, PP, PA6, and PET across CMP, PMP, and aged CMP sources, with mean values (●), medians (─), interquartile range (25-75%), and minimum-maximum values presented. Ten individual spike samples were measured (n=10).

**Figure S6.** ATR-FTIR spectra of micron-sized (A) PE, (B) PP, (C) PA6, (D) PET from CMP, PMP, and Aged-CMP.

**Figure S7.** MDSC thermogram of extracted CMP mixture of PE, PP, PA6, and PET from the soil matrix.

**Figure S8.** Correlation of TGA- and MDSC-determined mass using a plastic mixture spiked in a blank biosolid. The reference line in the plot is y = x.

**Figure S9.** TGA thermograms of environmental biosolid (A) sample 1, (B) sample 2, (C) sample 3. The black dot line represents the mass loss during the heating, and the red solid line represents the first derivative of the mass loss curve, smoothed with an 8-point window.

**References**

1. D. Sorolla-Rosario, J. Llorca-Porcel, M. Pérez-Martínez, D. Lozano-Castelló, A. Bueno-López, Study of microplastics with semicrystalline and amorphous structure identification by TGA and DSC, J. Environ. Chem. Eng. 10 (2022) pp. 106886. <https://doi.org/https://doi.org/10.1016/j.jece.2021.106886>.
